# Temperature-Stress Resistance and Tolerance along a Latitudinal Cline in North American *Arabidopsis lyrata*

**DOI:** 10.1371/journal.pone.0131808

**Published:** 2015-06-25

**Authors:** Guillaume Wos, Yvonne Willi

**Affiliations:** Institute of Biology, Evolutionary Botany, University of Neuchâtel, Neuchâtel, Switzerland; Chinese Academy of Sciences, CHINA

## Abstract

The study of latitudinal gradients can yield important insights into adaptation to temperature stress. Two strategies are available: resistance by limiting damage, or tolerance by reducing the fitness consequences of damage. Here we studied latitudinal variation in resistance and tolerance to frost and heat and tested the prediction of a trade-off between the two strategies and their costliness. We raised plants of replicate maternal seed families from eight populations of North American *Arabidopsis lyrata* collected along a latitudinal gradient in climate chambers and exposed them repeatedly to either frost or heat stress, while a set of control plants grew under standard conditions. When control plants reached maximum rosette size, leaf samples were exposed to frost and heat stress, and electrolyte leakage (PEL) was measured and treated as an estimate of resistance. Difference in maximum rosette size between stressed and control plants was used as an estimate of tolerance. Northern populations were more frost resistant, and less heat resistant and less heat tolerant, but—unexpectedly—they were also less frost tolerant. Negative genetic correlations between resistance and tolerance to the same and different thermal stress were generally not significant, indicating only weak trade-offs. However, tolerance to frost was consistently accompanied by small size under control conditions, which may explain the non-adaptive latitudinal pattern for frost tolerance. Our results suggest that adaptation to frost and heat is not constrained by trade-offs between them. But the cost of frost tolerance in terms of plant size reduction may be important for the limits of species distributions and climate niches.

## Introduction

The distribution of species may be determined in part by their ability to withstand sources of abiotic and biotic stress that vary clinally [1,2]. Indeed, evidence suggests that abiotic stress imposes strong selection along two widely-studied gradients—latitude and elevation—because populations are often locally adapted to changing thermal conditions along these gradients [3]. Thermal adaptation manifests itself in varying degrees of resistance to, or tolerance of, extreme temperatures. These two traits have rarely been studied simultaneously in the context of latitude mainly reflecting a temperature cline, and their fitness costs have rarely been assessed.

Life history theory predicts that coping with stress is likely to entail costs in other traits related to fitness [4,5]. These costs—referred to as trade-offs—may be important in determining species distribution limits. One trade-off that has been predicted theoretically is that between resistance and tolerance to the same type of stress [6,7]. Resistance is defense that prevents damage or limits its extent, whereas tolerance is defined as defense against stress that reduces the negative fitness impact of damage [8]. In the context of thermal stress in plants, resistance includes reduction in the degree of cell membrane injury [9]. Tolerance reflects the extent to which a plant maintains reproductive output despite sustaining damage from stress. Models of the joint evolution of resistance and tolerance suggest that the two may be alternatives; selection should maximize either strategy, but not both [10]. This is because the benefits of resistance are unnecessary in a highly tolerant individual, and the benefits of tolerance are rarely realized in a highly resistant individual [11]. Maximization of both traits therefore confers limited benefits while causing greater fitness costs than having either resistance or tolerance. This creates a trade-off between resistance and tolerance.

The resistance-tolerance trade-off has been well studied empirically in the context of biotic stressors [10,12]. While the idea that plants respond to biotic enemies with resistance and tolerance has been generally accepted, there is little empirical support for the prediction that the two strategies are negatively correlated. In fact, studies on plant-insect, plant-mammal, and plant-virus systems have often failed to detect phenotypic or genetic trade-offs between resistance and tolerance [13–16]. Instead, plants allocate resources to both strategies and the two can be maintained simultaneously at intermediate levels. Plant-thermal stress responses have rarely been studied in the context of life history evolution and trade-offs. This may stem from the difficulty of assessing tolerance against static stress factors without confounding it with the effect of resistance. For such stress factors—unlike, for example, for herbivory—it is hard to ensure that damage is equal across replicates. Agrawal et al. [17] measured selection acting on resistance and tolerance to frost in *Raphanus raphanistrum* in an outdoor garden experiment, and found that resistance was favored while tolerance was disfavored, most likely due to fitness costs of the latter. However, there was no genetic correlation between resistance and tolerance.

Other trade-offs important for species distribution limits may exist. Resistance and tolerance to different temperature extremes may trade off against one another. Furthermore, thermal-stress resistance and tolerance might trade off against components of fitness in the absence of stress, in which case we refer to costs of resistance/tolerance [18]. And finally, they might trade off against responses to other kinds of stress. Any of these trade-offs could become important for limiting adaptation if selection acting under a current temperature regime is perpendicular to a strong correlation between traits [19,20]. Limits to adaptation may also change with latitude. A species may be well-adapted to conditions at the center of its distribution, yet experience increasing fitness costs of adaptations to coping with increasingly stressful conditions towards the range margins [21]. In general, if adaptive strategies and fitness share a common genetic basis, trade-offs have the potential to limit the optimization of resistance and tolerance and hence species expansion.

In this study, we describe the latitudinal patterns in resistance and tolerance to frost and heat and the genetic trade-off between them within populations of *Arabidopsis lyrata* ssp. *lyrata*. *Arabidopsis lyrata* is a short-lived perennial, herbaceous plant closely related to *A*. *thaliana*. The species occurs in eastern North America from North Carolina to New York and in the midwest from Missouri to southwestern Ontario [22]. Eastern and midwestern populations form two ancestral genetic clusters; within these clusters the species has a fragmented distribution and most populations are genetically well isolated from one another [23]. The latitudinal cline is strongly correlated with mean annual temperature (higher temperatures in the south; [24]). This temperature gradient may create a gradient in natural selection, leading to a latitudinal cline in anatomical and physiological adaptations to temperature [25]. Thus, *A*. *lyrata* ssp. *lyrata* is an appropriate organism for assessing latitudinal gradients in resistance and tolerance to temperature.

**Fig 1:**
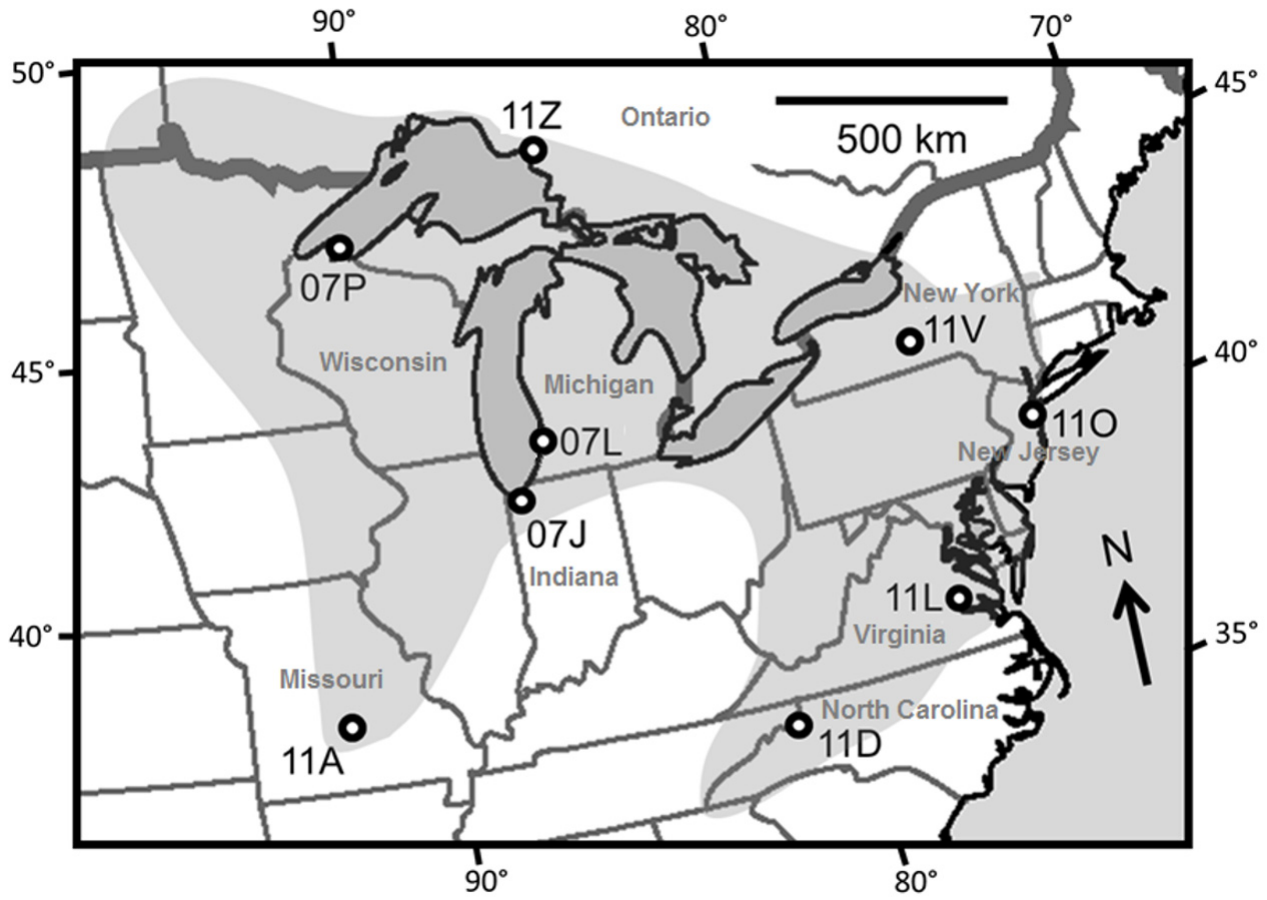
Locations of the nine North American *Arabidopsis lyrata* populations included in this study. The grey shading indicates the approximate distribution of the species based on herbarium records, regional botanical lists, personal communication with local botanists, and our own field experience. The actual distribution is highly fragmented. The eastern and western regions represent distinct ancestral genetic clusters [23].

In two climate chamber experiments, we assessed resistance and tolerance to frost and heat stress in plants of several seed families from each of multiple populations. We defined resistance as the difference in percentage electrolyte leakage between excised leaves that experienced no stress and leaves that were frost- or heat-stressed (analogous to [9]). Electrolyte leakage is caused by cell damage and therefore this method reflects cell membrane stability under stress [26]. Tolerance was defined as the difference in size between plants treated to regular frost or heat stress and plants experiencing control conditions. Size serves as an indicator of individual fitness in this analysis, which is reasonable for this species because the total size of the plant is correlated with reproductive output (see below). This measure of tolerance may not be completely independent of resistance; some plants may have continued growing well under the stress treatment because they were stress resistant. We addressed the following questions: (1) Do resistance and tolerance to frost and heat co-vary with latitude? (2) Does a negative genetic correlation exist between thermal-stress resistance and tolerance to the same temperature stress, and to different temperature stress? (3) Does a negative genetic correlation exist between thermal-stress resistance or tolerance and plant performance under no stress? Our first experiment compared populations along two parallel latitudinal gradients of 6 and 10° from North Carolina to New York and from Missouri to Ontario (S1 Table, Fig 1). Family means from this experiment were used to indicate the pattern of genetic correlations. A second experiment included many replicate families from one *A*. *lyrata* population to provide stronger estimates of genetic correlations observed in the first experiment.

## Materials and Methods

### Plant material

For *Experiment 1*, seeds of eight North American *Arabidopsis lyrata* ssp. *lyrata* populations were collected in 2007 and 2011. The sample included four populations near the northern and southern edges of the species’ distribution, and four that are more centrally located (Fig 1). In each population, several fruits of maternal plants were sampled over an area of about 500 m^2^; we used seeds of three maternal lines per population for this study. For *Experiment 2*, seeds of 40 plants were collected in 2010 from an area of about 2.5 ha in one population at Saugatuck Dunes State Park, Michigan, USA (42° 42′ N, 86° 12′ W; population 07L in Fig 1). All populations were outcrossing, judging from their low inbreeding coefficients [23,27]. Collection permits were granted by: Fort Leonard Wood Army Base; Michigan Department of Natural Resources; Rock Island Lodge, Michipicoten, Ontario; US National Park Service; Virginia Department of Conservation and Recreation.

### Experimental design

Field-collected seeds were raised in growth chambers and exposed regularly to three temperature treatments: frost, heat and control. The difference in final plant size between stress treatment and control was used as an estimate of tolerance. We measured resistance by excising leaf disks from control plants and measuring electrolyte leakage after exposing them to frost, heat or control conditions.

#### Experiment 1

The experimental design involved three spatially separated blocks, with one replicate plant per maternal line-treatment combination per block (8 populations x 3 maternal lines x 3 plant-growth treatments x 3 replicates/blocks = 216 plants). Seeds were haphazardly selected and sown in individual pots (dimension: 7 cm diameter, 5 cm depth; substrate 1:1 sand:peat). Pots were arranged in randomized positions on three holding trays per block. We placed two seeds into each pot, to ensure that we had at least one seedling per pot. Seeds were stratified for one week at 4°C in dark and kept under wet conditions. Trays were transferred into a growth chamber (Grobank, CLF, Germany) for two weeks during germination (18°C; 8 h:16 h light:dark; light intensity: 150 μmol m^-2^ s^-1^; relative humidity, RH: 40–70%). We increased the humidity around the seeds by covering pots with a perforated plastic cloth. At the end of the germination period, 78% of seeds had germinated and 184 pots had at least one seedling. Seedlings were haphazardly thinned to one per pot; some were transplanted into pots with no germinated seeds, so that there were 194 pots with seedlings in the end. Day length was then changed to 12 h:12 h light:dark (20°C:18°C day:night; light intensity: 180 μmol m^-2^ s^-1^; RH: 40–70%).

#### Experiment 2

The experimental design involved three replicates of each maternal line and treatment, arranged in three blocks (40 maternal lines x 3 plant-growth treatments x 3 replicates/blocks = 360 plants). Sowing procedure and conditions for germination were as in Experiment 1. At the end of the germination period, 81% of seeds had germinated. After thinning and transplantation, 353 pots had one seedling.

### Treatment during growth to assess temperature-stress tolerance

To assess tolerance to temperature stress, we exposed plants to one of three treatments during growth: control, frost and heat. Temperatures were chosen based on their relevance in nature. Frost events are not uncommon during the early growth period in spring—from April to May—in most of the locations where *A*. *lyrata* grows (S1 Table). Afternoon summer temperatures can reach 46°C near the basal rosette in mid-latitude populations (Y. Willi, unpublished data). Treatments were applied two weeks after the end of the germination period, when 80% of the plants were at the 4-leaf stage. In the frost treatment, plants were exposed to frost (-3°C) at the end of nighttime on three days in succession on each of three successive weeks. On each of these treatment days, we gradually decreased the temperature during the night. Starting at 18°C in the growth chamber, plants were transferred into a smaller cabinet (Sanyo electric Co., Ltd, Japan, model MLR-351H) with humidity conditions as in the growth chamber, then to a freezer, back to the cabinet, and then returned to the growth chamber. Temperature was decreased and then increased in steps of 1 h, starting at 18°C, then 0°C, -3°C, 0°C and back to 18°C. We followed a similar schedule for the heat treatment, except that plants were transferred at midday into a cabinet with similar light and humidity conditions as in the growth chamber. Temperature was increased and then decreased in steps of 1 h, starting at the base temperature of 20°C, then 30°C, 46°C in *Experiment 1* / 47°C in *Experiment 2*, 30°C and back to 20°C.

#### Growth trajectory parameters

The growth trajectory of plants was estimated from photographs of every holding tray made once a week for five weeks beginning at the end of germination. On each week, we measured the length of the two longest leaves of each plant using the software ImageJ v1.45s [28]. We selected an appropriate growth model for our plants by fitting seven alternative models to the mean leaf length for all weeks, separately for each plant. The models were: (1) linear, (2) exponential, (3) power function, (4) three-parameter logistic, (5) two-parameter logistic, (6) Gompertz and (7) von Bertalanffy. Models were fit in R version 3.0.1 (R Core Team 2013) with the package drc [29]. For both experiments, the best-supported model was the three-parameter logistic, which had the lowest Akaike information criterion value. In *Experiment 1*, average AIC weights for the 7 models were: (1) 0.0023, (2) 0.0214, (3) 0.0341, (4) 0.4975, (5) 0.0222, (6) 0.4217, and (7) 0.0004. In *Experiment 2*, average AIC weights for the 7 models were: (1) 0.0142, (2) 0.0231, (3) 0.0596, (4) 0.4414, (5) 0.1387, (6) 0.3162, and (7) 0.0067. The parameters of the three-parameter logistic model, estimated separately for each plant, are the asymptotic leaf length at the end of the growth period, the scale parameter, and x_mid_ (time until 50% of size is reached). The scale parameter is the inverse of maximum growth rate, *r*, so a large value corresponds to a low rate of growth. Parameter estimates for one plant in *Experiment 1* and for two plants in *Experiment 2* were discarded because they were >5 SD away from the mean; for asymptotic size, the direct measure from the last picture was taken instead. We also counted the number of leaves at the end of the experiment as a fourth measure of plant performance.

#### Calculating tolerance

Tolerance was calculated as the value of asymptotic rosette size of the stressed plant minus that of the control plant of the same maternal family within a block. We used asymptotic size as a measure of plant performance because it is strongly related with the number of fruits in the European sub-species of *A*. *lyrata* ssp. *petraea* [30] and with number of flowers in subsp. *lyrata* [31].

### Temperature-stress resistance

Resistance to stress in the absence of acclimation came from measures of percent electrolyte leakage (PEL) five weeks after the end of germination on plants growing under control conditions only. PEL measured on freshly collected leaves that are exposed to thermal stress or control conditions for some time reflects direct cell damage [32]. We picked the fifth and sixth rosette leaves from each plant and excised from each leaf three 5-mm diameter fragments. Leaf fragments were gently shaken in de-ionized water for 10 min to remove electrolytes from the surface, dried on a tissue, and then fully submerged in separate 1.5 ml tubes with 200 μl of de-ionized water. We applied one of three treatments to each tube: (1) control: incubation at 20°C for 1 h; (2) frost stress: incubation at -16°C in *Experiment 1* and -14°C in *Experiment 2* for 1 h in a freezer; (3) heat stress: incubation at 46°C in *Experiment 1* and 47°C *in Experiment 2* for 1 h in a water bath. Incubations were conducted in darkness. The heat temperature was the same as that applied to whole plants during growth. Both temperatures were selected based on preliminary experiments over a wide range of temperatures. After incubation, the leaf fragments rested at room temperature for one hour, after which conductivity of the solution was measured (Conductivity meter FE30—FiveEasy Mettler Toledo). Tubes were then placed in a boiling bath for 30 min and conductivity was measured a second time. PEL was conductivity after treatment relative to conductivity after the boiling bath in percent [32]. Resistance was calculated separately for each leaf as PEL of the control disc minus PEL of the stressed disc; low PEL values correspond to low damage, and low differences correspond to low resistance.

### Statistical analysis

#### Latitudinal variation

We first tested for latitudinal differences in growth parameters, number of leaves and PEL by hierarchical mixed model analysis using restricted maximum likelihood (PROC GLIMMIX, SAS Institute, 2006, 2008). Random effects were plant nested within family and population on the first level, family within population on the second level, and population on the third level; for the analysis of PEL, there was one lower level, the leaf nested within plant, family and population. Treatment was a fixed effect on the level of the plant, and block, ancestral cluster and latitude were fixed effects on the level of the population. Latitude was centered to a mean of 0. Similarly, we tested for latitudinal differences in resistance and tolerance to frost and heat stress with mixed models in which random effects were plant (resistance) or plant pair (tolerance) nested within family and population on the first level, family within population on the second level, and population on the third level. Again, for resistance there was one lower level, the leaf nested within plant, family and population. Block, ancestral cluster and latitude were fixed effects on the level of the population. In both kinds of models, interaction terms of cluster-by-latitude and cluster-by-latitude-by-treatment were never significant (*P* > 0.2) and not included in the final models. Latitude is a good proxy for temperature in this region: both mean minimum temperature during spring and mean maximum temperature during summer were strongly negatively correlated with latitude (mean minimum temperature March-May: *N* = 8, *r* = -0.90; mean maximum temperature June-August: *N* = 8, *r* = -0.73; monthly means from www.worldclim.org).

#### *C*orrelations between resistance, tolerance and plant size

A second analysis estimated within-population genetic correlations among resistance, tolerance, and plant size under control conditions across all populations. Family means were taken to reflect genotypic values. For Experiment 1, we standardized family means by population (mean = 0, SD = 1) and calculated Pearson correlation coefficients. For Experiment 2, we calculated Pearson correlation coefficients on untransformed family means.

## Results

### Latitudinal variation

#### Percent electrolyte leakage, PEL

**Fig 2:**
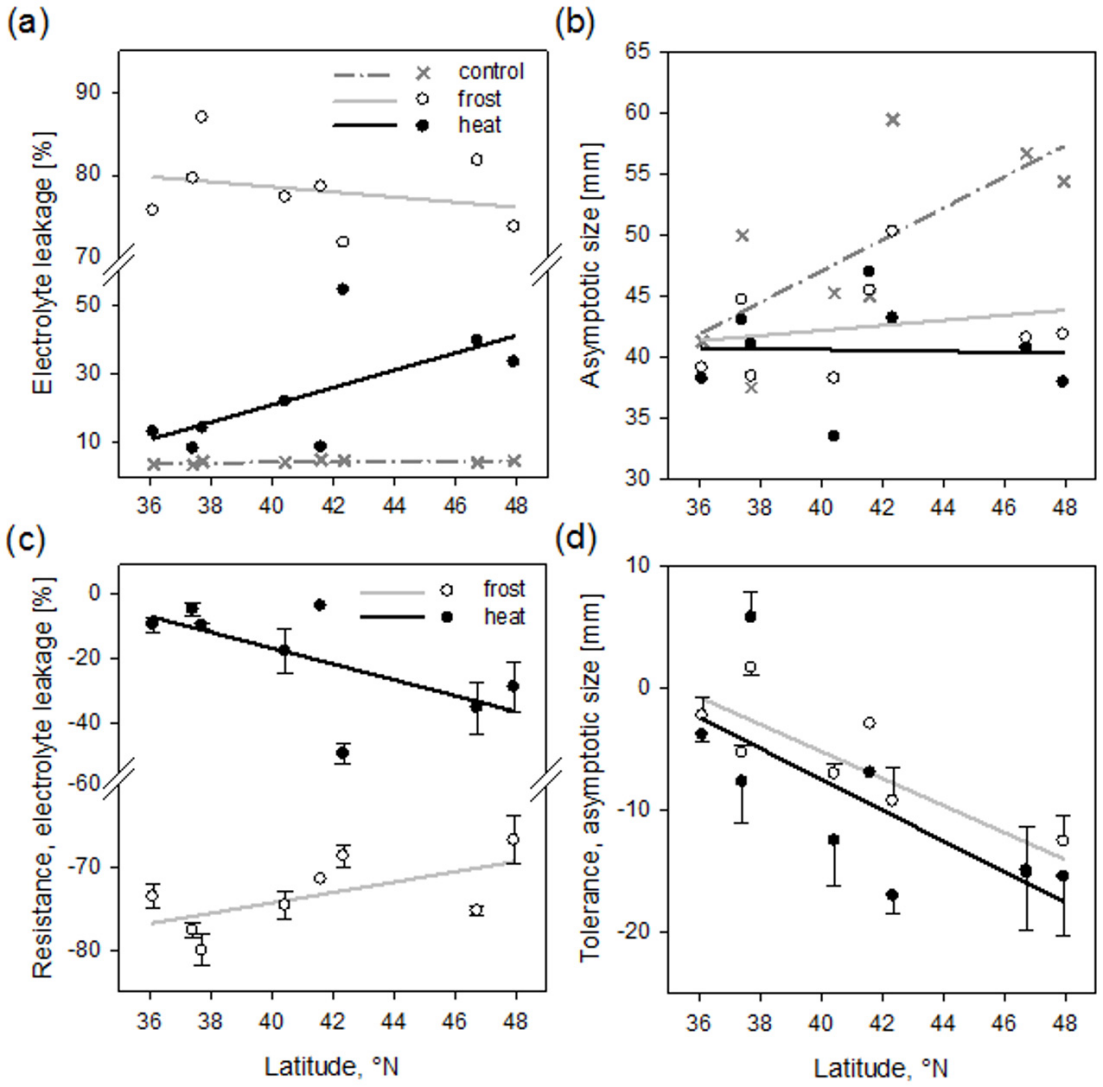
Latitude of origin of *Arabidopsis lyrata* plants differing in electrolyte leakage (a), asymptotic size (b), resistance to frost and heat based on electrolyte leakage (c), and tolerance to frost and heat based on asymptotic size (d). Symbols depict population means based on family means and one-/two-sided bars indicate standard errors. Regression lines on panels a and b represent the significant or close to significant latitude-by-treatment interaction, regression lines on panels c and d represent significant latitude effect. For statistics see Tables 1 and 2. Data for heat tolerance and frost resistance had been corrected for ancestral cluster.

Electrolyte leakage did not vary with latitude or ancestral cluster, but increased when leaves were exposed to stressful treatments (Table 1). PEL under control conditions was significantly lower than under frost and heat (least squares means, LSM ± SE control: 4.41 ± 0.39%, frost: 78.07 ± 1.74%, heat: 24.80 ± 4.70%). The treatment effect tended to interact with latitude: while PEL was about the same across latitude for the control treatment, it increased with latitude under heat, and decreased slightly with latitude under frost stress (Table 1, Fig 2a).

**Table 1:** Results of hierarchical mixed model analysis testing the effect of block, ancestral cluster, latitude, treatment and the interaction between the latter two on percentage electrolyte leakage (PEL), three parameters describing plant growth (asymptotic size, scale parameter and mid-point of growth x_mid_), and the number of leaves of *Arabidopsis lyrata* plants (*N* = 384, 194, 193, 193, 194).

			PEL		Asymptotic size	Scale parameter	x_mid_	Number of leaves
Dependent variables	df_num_	df_den_	*F*	df_den_	*F*	*F*	*F*	*F*
Block	2	14	0.49	14	3.54(*)	0.19	1.21	1.11
Ancestral cluster	1	5	0.22	5	1.21	0.37	0.37	2.53
Latitude	1	5	3.27	5	2.97	0.05	0.14	0.73
Treatment	2	14	855.90***	14	21.88***	0.34	0.16	3.80*
Latitude x treatment	2	370	2.84(*)	179/180	8.17***	1.59	0.25	2.33
		df	*t*	df	*t*			*t*
Frost vs. control		14	41.22***	14	-5.37***			-2.61*
Heat vs. control		14	4.32***	14	-5.94***			-2.30*

The table shows *F* values; the last two rows show *t* values for contrasts between pairs of treatments. Statistics for the random effects are not shown. Significance is indicated in bold:

^(^*^)^ *P* < 0.1,

*P < 0.05,

***P < 0.001

#### Plant growth

The asymptotic size of plants differed significantly among treatments but not with latitude or ancestral cluster (Table 1). Size was significantly smaller in plants growing in frost and heat treatments than in the control treatment (LSM ± SE control: 49.32 ± 1.72 mm, frost: 42.32 ± 1.63 mm, heat: 39.87 ± 1.88 mm). This indicates that frost and heat were stressful to the plants. The latitude-by-treatment interaction was significant because plants originating from higher-latitude sites grew to a larger size than low-latitude populations under control conditions, while under stressful conditions there were no trends with latitude (Table 1; Fig 2b). The other two parameters of logistic growth, the scale parameter and x_mid_, were unaffected by treatment or latitude (Table 1). The number of leaves at the end of the experiment was reduced in plants exposed to frost or heat stress, but was not significantly related to latitude (LSM ± SE control: 18.2 ± 0.9, frost: 16.6 ± 0.8 mm, heat: 16.8 ± 0.8 mm).

#### Temperature-stress resistance and tolerance

Resistance to frost and heat—calculated based on PEL—varied significantly with latitude, but in opposite directions (Table 2, Fig 2c). Populations from the north were more frost resistant and populations from the south were more heat resistant. Frost resistance was greater in western populations than eastern populations. (LSM ± SE western populations: -69.96 ± 1.38%, eastern populations: -77.83 ± 1.51%). Tolerance to frost and heat based on asymptotic size declined significantly with latitude (Table 2, Fig 2d). Populations in the western cluster were less tolerant to heat than eastern populations (LSM ± SE western populations: -14.54 ± 1.75 mm, eastern populations: -4.15 ± 2.20 mm). Results were unchanged when tolerance was standardized by the size under control conditions ([size stress—size control]/size control).

**Table 2:** Results of hierarchical mixed model analysis on resistance to frost and heat assessed from percentage electrolyte leakage (PEL), and tolerance to frost and heat stress assessed from asymptotic size AS.

				Resistance,PEL	Tolerance,AS
Stress	Dependent variables	df_num_	df_den_	*F*	*F*
Frost	Block	2	14	**3.15** (*)	1.58
	Ancestral cluster	1	5	**12.56** *	3.89
	Latitude	1	5	**11.56** *	26.60**
Heat	Block	2	14	0.17	0.85
	Ancestral cluster	1	5	2.41	**10.66** *
	Latitude	1	5	8.62*	**32.21** **

Sample sizes were 128 *Arabidopsis lyrata* plants for resistance, and 64 and 61 plant pairs for tolerance to frost and heat, respectively. The table shows *F* values for the fixed effects of block, ancestral cluster and latitude. Statistics for the random effects are not shown. Significance is indicated in bold:

^(^*^)^ *P* < 0.1,

* *P* < 0.05,

**P < 0.01

### Correlations between resistance, tolerance and plant size

#### Experiment 1

Genetic correlations between resistance and tolerance to the same type of stress were not significant (Table 3). This suggests no genetic trade-off between the two. The fact that the correlations were also not significantly positive suggests that our measure of tolerance was not strongly affected by resistance. Across stress types, resistance and tolerance to frost tended to trade off against heat resistance. Costs of resistance and tolerance, measured as genetic correlations with plant size in the absence of stress, were important only for frost tolerance. Relatively frost-tolerant genotypes were also relatively small. Frost resistance varied positively with plant size.

**Table 3:** Correlations between frost/heat resistance (RES_PEL_), frost/heat tolerance based on asymptotic size (TOL_AS_), and performance based on asymptotic rosette size of control plants of *Arabidopsis lyrata* (AS_control_) for two experiments.

			Frost		Heat		Performance
*Experiment*			RES_PEL_	TOL_AS_	RES_PEL_	TOL_AS_	AS_control_
*1*	Frost	RES_PEL_	1	-0.25	-0.42(*)	0.05	0.54*
		TOL_AS_		1	-0.37(*)	0.02	-0.50*
	Heat	RES_PEL_			1	0.02	-0.20
		TOL_AS_				1	-0.09
*2*	Frost	RES_PEL_	1	0.22	0.45**	0.22	-0.34*
		TOL_AS_		1	0.24	0.61***	-0.77***
	Heat	RES_PEL_			1	0.05	-0.18
		TOL_AS_				1	-0.67***

Correlations were performed on family means from eight populations (*Experiment 1*; population-scaled family means, *N* = 20–21) and on family means from one population near the distribution center (*Experiment 2*; *N* = 39–40). Significance is indicated in bold: (*)*P* < 0.1,

*P < 0.05,

**P < 0.01,

***P < 0.001

#### Experiment 2

Genetic correlations between resistance and tolerance to the same type of stress were not significant (Table 3). Across stress type, there were significant positive correlations between frost and heat resistance, and between frost and heat tolerance. Costs of frost tolerance were also detected, this time along with costs of frost resistance and heat tolerance.

## Discussion

Populations of *Arabidopsis lyrata* distributed across two latitudinal clines differ in several traits related to life history and persistence under thermal stress: plant size and resistance and tolerance to frost and heat. Plants from northern populations grew larger under control conditions, were more frost resistant, but less heat resistant and less heat tolerant compared to plants from southern populations. Surprisingly, plants from northern populations were also less tolerant to frost. We also found no evidence for a genetic trade-off between resistance and tolerance for the same type of thermal stress; nor was there consistent evidence for genetic trade-offs between resistance and tolerance for different types of stress. Resistance and tolerance to thermal extremes carried no consistent measurable costs, except that frost tolerance traded off against plant size under control conditions. The latter correlation was due to within-population variation.

The common garden design of this study emphasized genetic contributions to population divergence and variation among seed families within populations. Maternal environmental effects cannot be entirely ruled out, but they seem unlikely to have strongly impacted results. In herbaceous plants, early life-cycle traits such as seed size have been shown to be affected by maternal environmental effects, while later-expressed traits are not significantly impacted [33]. In *A*. *lyrata*, we have found that seed size is not correlated with a variety of later traits such as rosette size, carbon isotope discrimination, leaf dissection, trichome density, stomata density and length, and flowering time [34]. A few empirical studies specifically investigated the effect of developing seeds or parental exposure to low and high temperature and its carry-over effect to the next generation. In one accession of *A*. *thaliana*, exposure of parents to warm (25°C) and cold (15°C) conditions during flowering and seed development influenced some performance traits in their offspring and the speed of recovery of photosynthesis after frost but not longer-term recovery [35]. In replicate accessions of *A*. *thaliana*, exposure of parents to heat stress (40°C) or control conditions during their vegetative growth phase for two generations did not influence final performance traits in their offspring when assessed under heat stress and control conditions [36]. For these reasons, we assume that variation among populations and seed families in this experiment is mostly genetic.

### Latitudinal variation

Latitudinal trends in plant size and thermal-stress resistance and tolerance suggest that selection differs along the latitudinal gradient. The alternative—genetic drift—seems unlikely to have driven genetic differentiation because drift is a random force and therefore cannot create systematic differences in expressed traits along environmental gradients. The fact that results were qualitatively the same for the eastern and western clusters strengthens this conclusion. Moreover, latitudinal variation in size is consistent with data from many other plant species [37]. Our results also agree with previous studies on *A*. *lyrata* ssp. *lyrata* [24] and ssp. *petreae* [38,39], for which common garden experiments reveal that plants from high-latitude populations grow to larger size. In contrast, *A*. *thaliana*, a close relative of *A*. *lyrata*, apparently exhibits reduced growth rate, asymptotic size and leaf number at high latitude [40]. In our study, the two parameters reflecting the speed of growth—the scale parameter and x_mid_—did not significantly vary with latitude. Larger plant size in northern populations of *A*. *lyrata* may be associated with a generally faster reproductive development [24], possibly in response to more adverse conditions and a shorter vegetation period in the north (i.e., counter-gradient variation; Conover et al. [41]).

Latitude represents a complex environmental gradient strongly associated with temperature. Populations exposed to different temperatures along the gradient are expected to evolve correlated differences in characters related to resisting or tolerating thermal extremes [3,42]. Indeed, we found good evidence for this. Low-latitude populations exhibited elevated resistance and tolerance to heat stress, and this is consistent with the temperatures they experience in nature. High-latitude populations had elevated resistance to frost stress, which agrees with the association between frost resistance and latitudinal in *A*. *thaliana*, both with and without prior acclimation [43,44]. Unexpectedly, we also found that tolerance to frost declined with latitude; this may be due to trade-offs between frost tolerance and other performance traits, as discussed later.

### Correlations between resistance, tolerance and plant size

Our data did not support the hypothesis that resistance and tolerance of the same type of thermal extreme trade off against one another, at least in Experiment 1 (analysis of family means across populations). The situation is similar in the study of plant-herbivore interactions, for which evidence of a trade-off between resistance and tolerance is limited [13,15]. Among stress types, we also found no significant evidence for a trade-off between resistance and tolerance. In Experiment 1, coping with frost tended to trade off against heat resistance. And in Experiment 2 (many more families from one population), frost and heat resistance and frost and heat tolerance were positively correlated. Overall, our results provide no indication of constraints on the joint evolution of (increased) resistance and tolerance.

Evidence for costs of resistance and tolerance expressed as reduced performance under benign conditions was inconsistent. Costs of frost resistance and heat tolerance were detected in the experiment on seed families from one populations, but not in the experiment on seed families in multiple populations. An exception here was frost tolerance, for which costs were observed in both experiments (assuming that plant size was costly). The cost of frost tolerance could be an important constraint on adaptation at the northern edge of the distribution. Evidence suggests that selection in the north favors large size and an early switch to sexual reproduction [24], and frost tolerance is presumably also beneficial in cold, northern environments. But the trade-off between them implies that the two cannot evolve adaptively in the same time. This result is based on within-population genetic variation, but it matches exactly the pattern of among-population variation for plant size and frost tolerance. Plants from the north were large under benign conditions but were less frost tolerant. Plants from the south grew to smaller size under control conditions and their frost tolerance was higher. Thus, one explanation for the northern distribution limit of *A*. *lyrata* may be the combination of a short vegetation period and frequent frosts that delay flowering.

## Conclusions

Our study is among the first to systematically investigate the relationship between resistance and tolerance in the context of thermal stress. The rate of reproductive development is a well known mode of adaptation to latitude in plants (reviewed in Paccard et al. [24]), and our results suggest that thermal-stress resistance and tolerance may be important as well. Although there was no evidence for trade-offs between resistance and tolerance to the same thermal stress, we did find that thermal adaptation may be constrained by adaptation to other stress factors, for example the length of the growth and reproductive season. Evolution toward larger size and early reproduction is prevalent in the north, but may be impossible to maintain under frost. Obviously, finding generalities in these patterns across species would be of great interest for many fields, including climate adaptation, understanding species distribution limits and global climate change.

## Supporting Information

S1 Table Locations of *Arabidopsis lyrata* ssp. *lyrata* populations of this study and average number of days with negative temperatures (Frost days) recorded for April and May over the last 10 years, from 2001 to 2011. Weather records from the closest weather station of the populations studied were downloaded from the National Climatic Data Center webpage (http://www.ncdc.noaa.gov/). For population 11O, data were obtained from the Sandy Hook station for the years 2001, 2005 and 2008–2011, and from the Long Branch Oakhurst station for the other years. For population 11Z, data were obtained from the Wawa Station for the years 2004, 2005 and 2006 and from a weather forecast website for the other years (http://www.wunderground.com/history/)
(DOCX)
