# Supplementary material for: Temperature-Stress Resistance and Tolerance along a Latitudinal Cline in North American Arabidopsis lyrata
Source: PLoS One. 2015 Jun 25;10(6):e0131808. doi: 10.1371/journal.pone.0131808 (PMC4482397; doi:10.1371/journal.pone.0131808)
Supplement: S1 Table — Weather records from the closest weather station of the populations studied were downloaded from the National Climatic Data Center webpage (http://www.ncdc.noaa.gov/). For population 11O, data were obtained from the Sandy Hook station for the years 2001, 2005 and 2008–2011, and from the Long Branch Oakhurst station for the other years. For population 11Z, data were obtained from the Wawa Station for the years 2004, 2005 and 2006 and from a weather forecast website for the other years (http://www.wunderground.com/history/) (DOCX) [file pone.0131808.s001.docx]

**S1 Table.** Locations of *Arabidopsis lyrata* ssp. *lyrata* populations of this study and average number of days with negative temperatures (Frost days) recorded for April and May over the last 10 years, from 2001 to 2011. Weather records from the closest weather station of the populations studied were downloaded from the National Climatic Data Center webpage (http://www.ncdc.noaa.gov/). For the population 11O, data were obtained from the Sandy Hook station for the years 2001, 2005 and 2008-2011, and from the Long Branch Oakhurst station for the other years. For the population 11Z, data were obtained from the Wawa Station for the years 2004, 2005 and 2006 and from a weather forecast website for the other years (http://www.wunderground.com/history/).

| **Population** | **Location** | **Lat. °N** | **Long. °W** | **Weather Station** | **Frost days April** | **Frost days May** |
| --- | --- | --- | --- | --- | --- | --- |
| 11D | North Carolina | 36.11 | 81.66 | Boone, NC | 7.45 | 1.72 |
| 11L | Virginia | 37.41 | 77.02 | Williamsburg 2 N, VA | 0.72 | 0 |
| 11A | Missouri | 37.72 | 92.05 | Waynesville 2 W, MO | 4.72 | 0.45 |
| 11O | New Jersey | 40.44 | 73.98 | Sandy Hook, NJ | 1.36 | 0 |
| 07J | Indiana | 41.60 | 87.19 | Indiana Dunes NAT LK, IN | 4.63 | 0.27 |
| 11V | New York | 42.35 | 76.39 | Ithaca Cornell University, NY | 12.45 | 3.63 |
| 07P | Wisconsin | 46.73 | 90.80 | Madeline Island, WI | 21.72 | 7.81 |
| 11Z | Ontario, Lake Superior | 47.93 | 84.85 | Wawa AUT | 24.18 | 11.72 |
